# User and Professional Aspects for Sustainable Computing Based on the Internet of Things in Europe

**DOI:** 10.3390/s23010529

**Published:** 2023-01-03

**Authors:** Vera Pospelova, Inés López-Baldominos, Luis Fernández-Sanz, Ana Castillo-Martínez, Sanjay Misra

**Affiliations:** 1 Department of Computer Sciences, Edificio Politécnico, Campus Universitario, Universidad de Alcalá, Ctra. Madrid-Barcelona Km 33,600, E-28805 Alcalá de Henares, Spain; 2 Department of Computer Science and Communication, Høgskolen i Østfold, B.R.A. Veien 4, Remmen, 1757 Halden, Norway

**Keywords:** IoT, implementation, human factors, Europe, survey

## Abstract

The commonly accepted definition of sustainability considers the availability of relevant resources to make an activity feasible and durable while also recognizing users’ support as an essential part of the social side of sustainability. IoT represents a disruption in the general scenario of computing for both users and professionals. The real expansion and integration of applications based on IoT depend on our capacity of exploring the necessary skills and professional profiles that are essential for the implementation of IoT projects, but also on the perception of relevant aspects for users, e.g., privacy, legal, IPR, and security issues. Our participation in several EU-funded projects with a focus on this area has enabled the collection of information on both sides of IoT sustainability through surveys but also by collecting data from a variety of sources. Thanks to these varied and complementary sources of information, this article will explore the user and professional aspects of the sustainability of the Internet of Things in practice.

## 1. Introduction

Sustainability is a paradigm for thinking about a future in which environmental, societal, and economic considerations are balanced in the pursuit of an improved quality of life: “meeting the needs of the present without compromising the ability of future generations to meet their own needs” [1,2]. Sustainable development is a core principle of the European Union and a priority objective for the Union’s internal and external policies [3]. The objectives of the EU are aligned with the ones of the United Nations expressed in the set of 17 sustainable development goals [4]. The Internet of Things (IoT), understood as “a network of things, with clear element identification, embedded with software intelligence, sensors, and ubiquitous connectivity to the Internet” [5] has been identified as a contributor to sustainability in general, but more specifically to some of the SDGs: e.g., SDG 11 for sustainable cities [6,7] or SGD 7 for clean energy [8,9]. Sometimes, the contribution of IoT to the goal of saving energy is seen as controversial as it generates large amounts of data to be processed and the corresponding energy consumption (which could always be optimized [10]) but the abundance of data also contributes to other sustainability goals such as one of sustainable cities. Undoubtedly, these debates on the balance of sustainable computing with IoT are linked to the general idea of Green IT that emerged many years ago [11]. However, our focus is on the contribution of IoT as an enabler of disruptive innovations that promote safe, secure, and environmentally friendly lives to people, trying to find additional empirical insights on how the true impacts of IoT on sustainability can be ensured through a successful implementation [12]. As some research works have highlighted the importance of IoT with smart cities (SCs) [13,14,15,16], we will focus a good part of our work on the study of IoT in the context of SC projects.

The studies mentioned above show the potential contribution of IoT to sustainability, but its successful implementation may be hindered by different factors. Sometimes, the pressure of the market for the adoption of IoT technologies poses serious challenges for the involved organizations. For example, computing projects for innovation and sustainable growth, especially in SMEs, are highly dependent on having educated human resources to effectively address the specific internal and external activities through the IoT [17]. These challenges require constant technological and managerial support and improvement of contextual conditions for projects. While information security and privacy are rather apparent points of concern [18], there are many more IoT-specific factors that need to be addressed for the successful implementation and actual generation of value. The analysis of the factors that are essential for success in IoT projects, environments, and initiatives is a prolific area in the literature. The related work has tried to formalize the impact of factors in IoT projects in the shape of maturity models [19]. These models have been found effective for, firstly, the assessment and, secondly, the improvement in this process by breaking it down into highly detailed steps [20]. These models help to analyze or even predict the success of initiatives by assessing the possible set of all or the most relevant influential factors. They also guide the efforts of organizations to reach the best conditions for success in IoT initiatives.

The number of maturity frameworks related to the different possible contexts of projects linked to IoT is high in the existing literature, frequently linked to the concept of Industry 4.0 [21]. As we want to explore both the side of users and that of professionals within solution providers, we will focus on the B2C context. The work by Klisenko and Serral [20] has analyzed 16 different models applicable to readiness for IoT in B2C, although considering different aspects of the area. As a result of the compilation and analysis of those models, two main human factors are identified, apart from other technical and organizational factors:

- The connection with customers for IoT adoption considering their attitude and fears toward this technology is also complemented by the culture of users: employees that will apply IoT solutions in their daily work. This aspect has been also identified, sometimes embedded in the culture of the organization, in additional studies such as [22,23,24];
- The capabilities of the IoT implementation support team, as this is an essential resource for success, are also identified in specific projects [22,25].

Another research work conducted by Brandstetter [26] demonstrated how new business models can be successfully implemented thanks to a transnational approach, which leads to close cooperation between different partners. Furthermore, a high number of EU projects are carried out transnationally to fulfill one of the European pillars: inclusiveness and cooperation [27].

This research is aimed at studying the two above-mentioned main human factors for IoT success: a) the attitude and culture of customers and users toward this technology and b) the qualification profile of the professional support team. Therefore, this study worked with two different surveys: the first one measures the impact of IoT on users and their attitude toward IoT projects and solutions, while the second one explores the recommended professional profile for a successful implementation of IoT in one specific context: smart cities (SCs) projects. Although restricting the IoT context to SCs would represent a relevant limitation, the process of this study has included additional sources of information to determine whether the results are applicable to most contexts where IoT is implemented. In the end, this second part of the research will explore the experts’ opinions to study the influence of new professional profiles on existing frameworks and models of project work.

This article is structured as follows: Section 2 presents the research questions of our study and the methodology for answering them, briefly defining the motivation and goals of the two surveys developed for the study. Section 3 presents and discusses the results of the survey on the impact factors for the implementation of IoT solutions according to users’ perceptions. Section 4 describes and discusses the results of the recommended profiles for a successful IoT implementation. Section 5 proposes conclusions and future research lines.

## 2. Methodology

The process starts with the following research questions as an expression of our research goals:

- RQ1: what are the key factors perceived by users and customers in Europe, within their scope of action, for effective IoT deployment?
- RQ2: which is the most recommended qualification profile for ICT professionals in Europe for an effective IoT implementation?

Our main method for answering RQ1 was the development of a specific survey on the factors that influence the success of the implementation of IoT solutions from the point of view of users and customers, also analyzing the possible implications for their professional or business activity. The answer to RQ2 required the exploitation of another survey, this time within the frame of an EU-funded project on SCs that explores the recommended professional profiles for the projects in this area. IoT plays a very relevant role in SC projects, so we analyzed the specific questions on the qualification of the technical team in this part of IoT. Both surveys are complementary and enabled the coverage of the two human-related factors identified in our analysis in Section 1. The next subsections will describe the design of both surveys.

### 2.1. Survey on Factors That Influence IoT Implementation from the Point of View of Non-Technical Professionals

IoT is bringing relevant and even disrupting transformations to very different productive and professional areas. Different works have confirmed the power of IoT for the transformation of business models and organizational models [28,29]. The impact of IoT not only reaches the professional and business side: several authors [30,31] have analyzed the impacts of IoT as a social transformer. Others [32] consider this topic as one with the highest priority. As a social transformer, IoT is frequently conditioned by legislation, which may differ from one country to another, so studies must adopt the multinational approach to be effective while analyzing both the effects in business and in society.

Losavio et al. [33] analyze data management laws protecting the rights of people in terms of privacy and security as well as the rights of personality and personal autonomy in different nations, relating IoT aspects to SC projects. The analysis shows how we need clear legislation on what can and cannot be done, balancing public security with individual freedoms across different countries. Not only is the evident case of legislation dependent on the country where IoT acts: research conducted by Zallio [34] collected information of users as feedback comments to increase the usability of IoT devices. The results demonstrated the importance of IoT-based devices in daily activities and relevant variations depending on the countries, cultures, and personalities of individuals. All these findings suggest that the analysis of the user side in IoT implementations should cover different countries (in our case, in the European Union) and should also explicitly inquire on the possible commonalities and differences among countries.

In general, transformations linked to solutions based on IoT tend to encourage social concerns and worries about safety and rights. Several works [16,17,20] have analyzed the challenges of privacy and security produced by IoT and proposed approaches to mitigate some of these fears that influence user’ and customer behaviors. Other authors have studied the users’ concerns regarding data privacy and security when they decide to purchase IoT solutions [22]: these worries impacted almost all the users surveyed. Therefore, we explicitly included a question on these aspects in our survey.

Regarding the educational approach, a research [35] analyzes the impact of IoT in different educational approaches. The study shows the effectiveness of establishing IoT-based learning frameworks, generating new paradigms of learning. Despite its importance, the study also determines several challenges for the inclusion of IoT in the curricula and highlights the importance of training all types of professionals, not only the ICT professionals. In fact, various studies have identified the impact of IoT on changes and challenges in the qualification of non-ICT professionals [36,37]. This is the reason why we also explore this aspect in our survey.

Motivated by the goal of exploring the above-mentioned aspects detected in previous studies, we surveyed to examine the impact of IoT on users and other relevant stakeholders involved in the implementation of solutions. We avoided addressing people with IT backgrounds, as the objective was the perception of those without specialization in IT. One additional goal was determining the need for training to help these people to adapt their business models and their daily work to a new context with IoT, while also examining the general social impact. This survey was designed to explore the best approach for engaging, training, or re-skilling all types of non-technical professionals to be prepared for a forthcoming massive implementation of IoT in their activity sectors and all aspects of life.

#### Design of the Survey

The first part of the survey was designed to identify the profile of the respondent. Age, years of working experience, and familiarity with IoT helps to identify the confidence of the user in the topic, while the sector and size of the working organization will allow for measuring the challenges of IoT for the organizations. The age of the respondents was measured in a group of 5 years starting from 25 until 64 years old, being less than 25 or more than 64 in a different group. The years of working experience, however, were measured in groups of 10. Regarding the familiarity with IoT topic, four possible options were presented, as none, basic, advanced, or professional experience. We also added the country to control the geographical variety of the sample. Free space for comments was also provided to the user to gather other relevant opinions (e.g., about the survey).

The objective of the next section of the survey was the exploration of the relevant impact factors in practical IoT implementation from the perspective of non-technical professionals. It was implemented as statements linked to the different areas above-mentioned in the previous section:

- Business models, marketing, and customer service: the transformation of business processes and new business models;
- Security, data privacy and protection, and IPR: the types, amount, and specificity of data gathered by billions of devices create concerns among individuals about their privacy and among organizations about the confidentiality and integrity of their data;
- Employment and qualifications: IoT would imply the need for upskilling and reskilling non-ICT professionals after a careful analysis of profiles and the requested hard and soft skills;
- Social and environmental aspects: IoT opens an opportunity to decrease the environmental impact of activities by avoiding physical presence and trips, reducing carbon footprint, and fostering more social balance.

The participants were asked to mark their level of agreement with 8 statements linked to the mentioned factors, expressed in a 5-level Likert scale: totally disagree, disagree, neither agree nor disagree, agree, totally agree.

- (S1) The adaptation to the impact and changes which IoT may bring to people, society, and businesses deserve the highest priority in all European countries;
- (S2) The impact and the implementation of IoT may differ from one to another country due to specific market conditions, legislation, etc.;
- (S3) Study and training of the adaptation to the impact of IoT recommend an international perspective for addressing different national views;
- (S4) Training all types of professionals in IoT literacy is essential for a successful and beneficial implementation of IoT in all sectors.

For a successful and beneficial implementation of IoT, the information and training on its changes and challenges are very important:

- (S5) In business models and market competition;
- (S6) In employment, occupation profiles, skills, and qualifications;
- (S7) In privacy, security, and legal consequences;
- (S8) In social aspects and transformations.

### 2.2. Survey on the Qualification of the Technical Team for the Successful Implementation of IoT

As detected in previous research on the SCs sector [38], with an intensive presence of IoT solutions, there is a deficit in the analysis and development of qualification profiles for the technical team responsible for solution implementations. In fact, the study of the SmartDevops project [38] finally determined three initial job profiles (“smart city planner”, “smart city IT manager”, and “smart city IT officer”) but mainly focused on the contribution of DevOps to the SC projects. Given the wide set of contexts where IoT implementation may happen, we have also exploited the SMACITE EU-funded project, also focused on SCs, [39] to more deeply explore what technical professionals need in terms of qualification referred to the implementation of IoT and in other complementary aspects such as security, data management, etc. The project reviewed the main educational references on SCs (degrees, masters, and non-official postgraduate programs) as well as five case studies provided by partners from Spain, Bulgaria, Belgium, and Greece. This information helped to detect the consideration of different groups of skills and knowledge that are vital for a qualified technical workforce for SC implementation. Thanks to this analysis, it was possible to identify different technical categories. Some of these categories (such as enabling technologies, management, and business, or green and soft skills) were similar to the ones determined by the above-mentioned research in contexts where IoT plays a prominent role. We exploited this context of SCs for IoT implementation to explore, with a survey, the most recommended qualification profile for ICT professionals in Europe for an effective IoT implementation (RQ2).

#### 2.2.1. Survey on Qualification Profile for Professionals Who Implement IoT Solutions

As part of the SMACITE project [39], we designed a survey to collect the opinion of a broad base of stakeholders to determine the recommended skills and knowledge profile for ICT professionals working in the context of SC projects, both at the engineer and technician level. Our interest was determining the recommended qualification profile for ICT professionals, mapping it to the two most relevant frameworks for technical occupations and job roles: ESCO [40], which is the European multilingual classification of skills, competences, qualifications, and occupations, and EN16234 [41], the European standard on e-competences for ICT professionals. The nature of e-CF is different from the one of ESCO:

- The normative part of EN16234 is focused on the description of the 41 e-competences in terms of the main functions and activities developed in each one;
- It also includes descriptions of levels of proficiency and examples of skills and knowledge items, but they are only illustrative.

Our analysis focused on the requirements of qualification in the field of IoT and other key aspects such as privacy, legal, IPR, security issues and data analytics, and machine learning. The survey was designed, as usual, with a first general part for collecting the basic profile of the respondent (country, age, years of experience, etc.). Then, follows a set of statements on functions and responsibilities in SC at the engineer and technician level plus a set of questions on the relevance of categories identified in previous research on technical skills (enabling technologies, management and business, green and soft skills). The next section of the survey also explored the recommended skills and knowledge items, taken from ESCO, for smart city technicians and engineers: their descriptions were presented as a compilation of the most relevant existing in the skills pillar of the classification connected to each category, to each enabling technology, or to similar occupations. The final shape of the statements was reviewed and selected by a focus group of seven experts from the partners participating in the project.

The last section focused on the recommended soft skills (non-technical skills) as they are very linked to success in effectiveness, professional performance and career [42,43]: this is confirmed by employers as they consider that soft skills are less trainable than technical skills, while the performance of a hard skill is often dependent upon soft-skill capacity [44]. As there is no widely accepted model of soft skills, we adopted one of the Skills Match projects [45,46] with a framework of 36 soft skills, also directly matched to ESCO [40].

## 3. Analysis of Results and Discussion on Users’ Perception of Factors for IoT Implementation

As commented in Section 2.1, the survey on users’ perception of IoT implementation was aimed at answering RQ1. The participation was targeted at non-technical professionals, especially those in SMEs, as these types of organizations have fewer resources to adapt their business models and daily activities to new paradigms. The survey was also addressed to those involved in the education and training of future professionals in non-IT fields.

### 3.1. Sample

Most of the 48 respondents to the survey were from European countries and other associated countries. The rate of participation was 37.5%, as there was finally a total of 128 clicks on the link. The participants who answered this survey were in majority from Ireland and Spain, followed by eastern countries such as Latvia and Bulgaria. The age of the participants was diverse and balanced: the highest proportion of age was 25–29 (17.6%), followed by 35–39 (15.7%), and 45–49 (13.7%). Regarding the years of working experience, respondents in the range of 1–9 years (33.3%) had the highest percentage, followed by those in the range of 20–29 (23.5%).

The working sector of the participants was diverse (up to 12 different ones), although the most frequent was IT (but respondents were not ICT professionals, only non-technical employees and managers) with 29% of responses and the second one was the education sector (25%). Other sectors such as marketing, engineering, management, etc., completed the sample, all under 9%. Regarding the size of the organization, most of the participants were working for SMEs (33%) with less than 250 employees, micro-SMEs (21%) with less than 10, and public education (21%). The rest of the participants worked for medium and big companies and the public sector of government.

Participants in the survey also self-rated their familiarity with IoT. Most of the users declared to have only basic concepts (54%), while one-third of them claimed to have advanced knowledge (31%). Only 11% had some professional experience in the area, while 6% had neither experience nor knowledge.

### 3.2. Analysis of Survey Results

**Figure 1:**
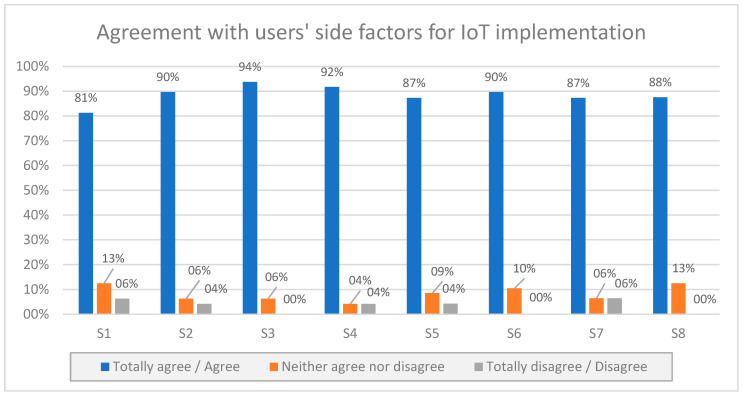
Summary of agreement of users with statements on impact factors for IoT success.

This section presents the results expressed as respondents’ agreement level for the statements S1–S8 presented in Section 2.1. The end of this section contains a chart summarizing the results. Respondents showed the following levels of agreement (see Figure 1):

- S1—IoT impact and changes in people, society, and businesses: most of the respondents agree (51%), while 31.4% totally agree, and 11.8% neither agree nor disagree;
- S2—Differences among countries in conditions for IoT implementation: 56.9% agreed and 33.3% showed total agreement, while the other available options were below 6%;
- S3—Transnational approach when analyzing IoT implementation: almost half of the respondents totally agree (49%) and another 45.1% also agree;
- S4—Training of all types of professionals in IoT literacy is essential: again, half of the respondents totally agree (49%), another 43.1% agree, and only 3.9% disagree;
- S5—IoT changes and challenges in business models and market competition: 39.2% totally agree and 47.1% agree but only 3.9% disagree;
- S6—IoT impact on employment, occupation profiles, skills, and qualifications: 47.1% totally agree and 43.1% agree. Neither agree nor disagree represented 9.8% of respondents;
- S7—IoT impact on privacy, security, and legal consequences: total agreement 47.1%, agreement 39.2%, and the rest of the options represented less than 6%;
- S8—IoT impact in social aspects: total agreement reached 51%, agreement 35.3%, while the option of neither agree nor disagree obtained 11.8%.

The free space left for comments in the survey only attracted three comments without relevance to the analysis.

The first conclusion is the high level of agreement of participants to the statements, something that surprised us. This means that our study confirms the findings from the preliminary review of the literature, although logically there are evident limitations in the size and the composition of the sample. There are no relevant and meaningful differences in percentages of agreement or total agreement when segmented by country, age, or experience. However, the size/type of organization shows some differences regarding the average percentage of agreement in the eight questions: micro-SMEs and education organizations are less convinced than the rest, while medium-size (although with a small sample) organizations show the highest values. In contrast, there are no relevant differences in the opinion of participants regarding the self-declared level of familiarity with IoT: only those with some professional experience with IoT (again, a small sample) show a slightly lower level of agreement.

However, the results suggest that the sample of EU non-technical professionals confirms that their adaptation to changes caused by IoT is key to success. It is also essential to their awareness of possible differences among countries due to non-homogeneous conditions or legislation in the different national contexts. In general, they also agree on the importance of training in two aspects: (1) adaptation to new contexts created by IoT implementation and (2) acquisition of basic IoT literacy skills. According to the results, the support should also be complemented with information and specific training in (a) changes and adaptation of business models, (b) occupation profiles, skills, and qualifications, (c) challenges in privacy, security, and legal consequences, and (d) changes and challenges in social aspects such as environmental effects and inclusion.

The survey has enabled a detailed answer to RQ1, generating a list of more specific points than the mere general description of the human factor described as “connection with customers for IoT adoption considering their culture and their attitude and fears towards this technology” mentioned by the literature (See Section 1). These details may help to adopt more effective actions for a successful and more sustainable implementation of IoT in the future.

## 4. Analysis of Results and Discussion on Recommended Profiles of the Technical Team for Successful IoT Implementation

As commented in Section 2.2, the second survey focused on the qualification of the technical team for IoT as part of the SMACITE project [39] and it provides information to answer RQ2. The participation in the survey was targeted to three different categories of stakeholders linked to SC projects: (a) the customer side, with municipal authorities, managers, and technicians, (b) the provider side, with managers and professionals from solution development companies, and (c) the user side, with representatives of citizens’ associations and independent experts. Although disseminated across Europe in English, some partners of the project translated it into local languages to facilitate participation in their countries: Spain, Italy, and Greece.

### 4.1. Sample

Project partners disseminated the online survey through different networks, specifically targeting contacts belonging to any of the categories of stakeholders. The rate of participation was 34%, as there finally were 134 contributions from a total of 394 clicks on the link.

The first section of the survey collected the basic profile of the country and gender from each of the contributors. The nationality of the respondents was diverse, with 11 European countries identified. The highest number of contributions came from Spain (34.07%), Greece (16.30%), Bulgaria (27.41%), and Italy (13.33%). Gender representation was unbalanced: 71.1% male, 26.6% female, and 2.2% preferred not to say their gender.

The stakeholder category included three main options with different sub-options as job roles: public sector and authorities (client side), business sector and providers (supply side), and civil society (user side). The sample contained 18.52% of participants from the group of public sector and authorities, 54.81% from solution providers, and 26.67% from civil society, user representation, and independent experts. Table 1 shows the distribution among roles of the three categories, showing the variety of roles (except for the case of “Municipal city planner or urbanism expert” without representation).

The years of professional experience are also important for analyzing the results: the answer options appeared in groups of five, with options for less than five years, steps of five between 5 and 20, and more than 20. The contributors mostly concentrated in more than 20 years (48.15%) and less than 5 years (15.56%), while all the rest of the options were under 11.11%. However, the general experience is not the only factor that may have an impact on opinions. We also requested participants to self-declare their familiarity with SC concepts and solutions on a scale with five options. The distribution of the sample was: none (5.19%), basic knowledge (30.37%), application of concepts out of professional practice (25.19%), professional experience in the area (28.15%), and highly qualified and experienced in the area (11.11%).

### 4.2. Analysis of Results on Qualification of the Technical Team

The main section of the survey collected the opinion of the participants on the set of functions, skills, and knowledge based on ESCO and e-CF [41] determined by our preliminary analysis (see Section 2.2.1). Participants were asked to rate each item according to their understanding of the relevance for recommending it for the qualification profile, for both engineer and technician roles. The questions adopted a 5-option Likert scale (essential, relevant, useful, marginal, worthless) plus a “not sure” option. The design of the description for each item was concise and synthetic, thus avoiding excessive time and effort: a focus group with experts from project partners generated descriptive phrases as a compilation of items inspired and extracted from specific ESCO occupations, selecting the ones with the highest conceptual similarity, and their skills and knowledge items.

An expert group with representatives of project partners performed an analysis of information through several methods for the identification of relevant skills and knowledge items in ESCO: on one hand, a direct search on the ESCO website using various keywords to obtain results related to SCs; on the other hand, a local replica of the whole database of the ESCO website allowed for a deeper search on skills and knowledge through specific sophisticated queries not possible on the website. This led to the identification of 15 ESCO occupations with relevance in the SCs context: “Smart home engineer”, “Smart home installer”, “Civil engineer”, “Civil engineering technician”, “Cloud engineer”, “ICT security engineer”, “ICT security technician”, “Data analyst”, “Data scientist”, “3D modeler”, “3D printing technician”, “Blockchain architect”, “Blockchain developer”, “Project Manager”, and “ICT Project manager”. Then, the expert group extracted 89 knowledge and skills from the descriptions of these occupations as the most relevant related set of items for the qualifications of technical professionals in SC projects.

Regarding the reference to the e-CF (EN16234) framework [41], the first analysis focused on the set of 30 examples of description of professional roles with their e-competences. However, none of these roles exhibit a reasonable degree of similarity in terms of functions or responsibilities to the target smart cities profiles. Moreover, these descriptions are also mere examples, not exhaustive descriptions, as the number of e-competences mentioned in each of them is limited to five on purpose. Therefore, the final approach with e-CF was working with the mapping of the resulting set of functions from ESCO occupations to link them to the equivalent e-competences and proficiency levels in the standard. The final set of e-competences comprised nine of them (B.6, E.2, A.6., B.4, E.8, D.7, B.3, B.1, and C.1) and different levels ranging from level 1 to 4.

#### 4.2.1. Functions for Engineers and Technicians

The proposed functions for the SCs engineer and the SCs technician came from the description of the different similar ESCO occupations as mentioned above (see Table 2 and Table 3 for details).

**Figure 2:**
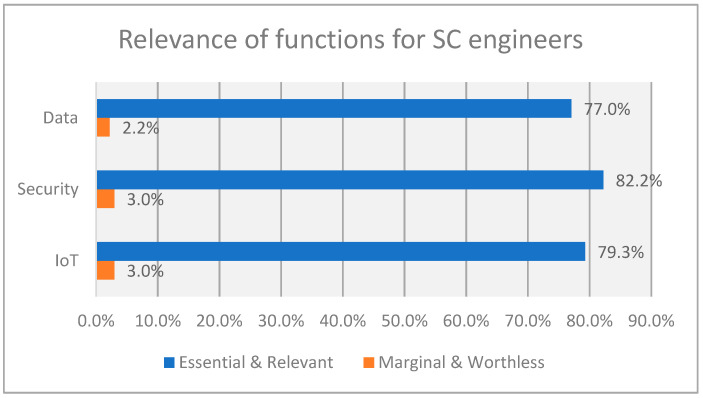
Summary of relevance of functions for SC engineers.

**Figure 3:**
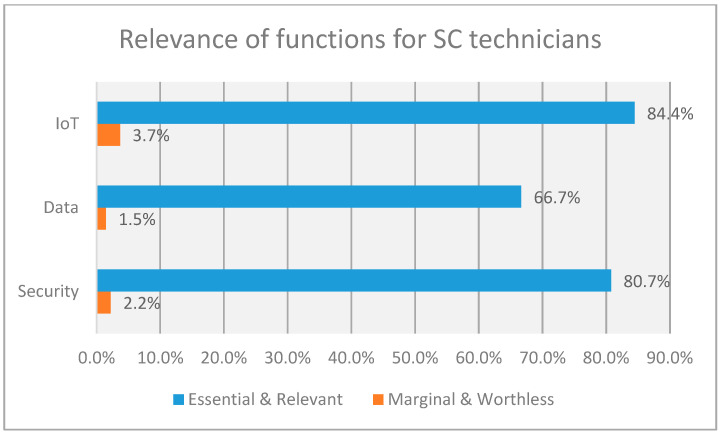
Summary of relevance of functions for SC technicians.

The results from the survey show the relevance of functions and responsibilities allocated by participants for the determination of the recommended profile of SC engineers and SC technicians (see Figure 2 and Figure 3). As we can see, the work in SC projects for engineers and technicians is intensive in IoT as well as security and data management. This confirms that the study of the data from our survey can be representative of the analysis of qualification for the implementation of IoT solutions in general, combined with the aspects of security and data management.

Once the profile of functions was determined, the development of the mapping to the EN16234 framework [41] mainly considered the equivalence of those functions with responsibilities and activities described in dimension two of the standard. The detected relations were very direct: only a small number of functions needed to be linked to two e-competences to ensure a correct representation of the activities. The final mapping developed by the expert group after analyzing the survey is shown in Table 4.

#### 4.2.2. Knowledge, Skills, and Soft Skills

In the case of skills and knowledge, the expert group of the project identified the most relevant skills in ESCO for the occupations already used for the functions (see Section 4.2.1). Table 5 and Table 6 show the selected ones, the most relevant for SC engineers and for technicians. The participants in the survey had to answer the question: “According to your experience, up to what extent is this skill/knowledge important for SC engineers/technicians?”. Table 5 shows the specific descriptions of skills and knowledge for an SC engineer, together with their inspirational basis from ESCO; Table 6 shows the one for an SCs technician. The description is a summary of the most meaningful features of the corresponding skills or knowledge items identified in ESCO.

In the case of soft skills, the statements for the survey were almost the same for engineers and technicians. The reference model was one of the Skills Match projects [45,46]. The model has 36 soft skills, but it also identified clusters of skills intimately linked among them. For the sake of simplicity, the survey referred to the relevance of the clusters for the profiles. The list of clusters of soft skills is the following one:

- Accountability (customer focus, diligence, reliability, efficiency);
- Communication (networking, negotiation, teamwork);
- Creativity (critical thinking, problem-solving, decision-making, initiative);
- Ethical behavior (respect diversity, respect environment, respect privacy);
- Leadership (coaching, conflict resolution, entrepreneurship, strategic thinking, motivating others, managing quality);
- Self-management (adaptability, organization, positive attitude, self-control, personal development);
- Tenacity (goal orientation, patience, motivation, resilience).

**Figure 4:**
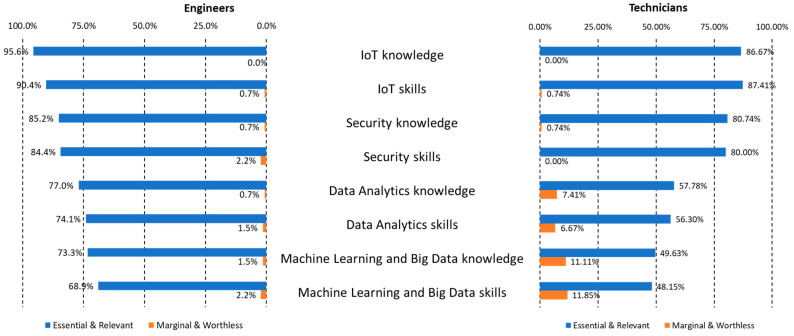
Summary of perceived relevance for skills and knowledge.

Figure 4 summarizes the opinion of the participants regarding the main categories of skills and knowledge. Both IoT knowledge and skills are the most recommended categories for the qualification of SC engineers and technicians. This represents another confirmation that our analysis focused on SC projects is practically equivalent to the one for the general implementation of IoT, suggesting that our conclusions can mostly be applicable to general IoT projects.

Cybersecurity is considered slightly less relevant than IoT but has equivalent high levels of agreement both for engineers and technicians. Data analytics skills and knowledge are the area in the third position but, in this case, while it is essential or relevant for engineers (74.1% in skills and 77% in knowledge), the values are considerably lower for technicians (around 57%). In this category, it is also possible to find a relevant proportion of disagreement, especially for the technician profile.

The area of Machine Learning and Big Data skills and knowledge represents the last relevant option in the ranking. Again, while it is most essential or relevant for engineers (around 70% for skills and knowledge), for technicians that consideration hardly reaches 50%. Clearly, this area is not considered a key factor for the qualification profile of SC technicians.

Apart from the evident descriptive results showing percentages of relevance, the data in Figure 3 and Figure 4 suggest a clear trend: the importance of skills over knowledge for the qualification of technicians, while knowledge is at the same level or even higher than skills in the different areas for engineers. The participants in the survey considered that the role of technician should be mainly focused on practical aspects while the engineers need more knowledge for their activities. The analysis of mapping to e-CF in Table 4 is also consistent with this idea of differences between technicians and engineers: functions for engineers exclusively relate to proficiency levels 3 and 4 of the standard, with high levels of influence within the organization, context complexity, and autonomy. Technicians are mainly linked to levels 1 and 2 (only the competence E.8, Information Security Management, reaches level 3), more connected to following instructions or applying and adapting procedures in structured and predictable contexts with limited independence or under general guidance.

**Figure 5:**
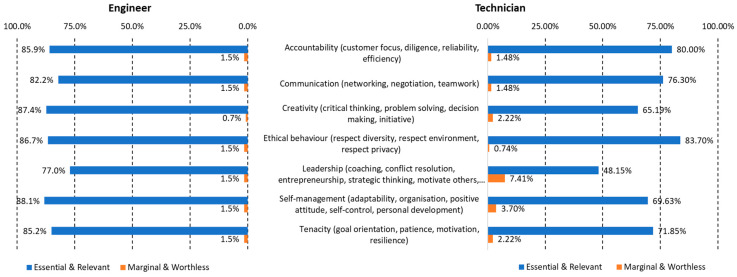
Summary of responses to the agreement on soft skill clusters.

Finally, regarding soft skills, every cluster was considered essential or important for engineers by at least 77% of the participants in the survey, with insignificant percentages for the options marginal and worthless (see Figure 5). In the case of technicians, there is a greater disparity of values. However, more than 50% of the participants considered all the proposed clusters of soft skills to be relevant, except for leadership with only 48% and creativity with a relatively high value of importance (65%). The results for these two clusters are consistent with the results for the e-CF mapping mentioned above, as low proficiency levels are linked to limited autonomy and work under guidance, mainly following instructions and procedures.

It is worth mentioning that the effort of developing the survey for RQ2 and the final mapping of the profiles to both ESCO and EN16234 has involved a considerable number of references to items from these models:

- Development of survey:
  ○ A total of 15 reference occupations selected from the 3008 existing in the version 1.1 of ESCO;
  ○ A total of 89 skills and knowledge items, connected to the 15 occupations, were selected from the total catalog of 13,890 in ESCO;
  ○ A total of nine e-competences from EN16234 linked to 11 functions for the profiles through 14 pairs of competencies and proficiency levels.
- Recommended profiles:
  ○ Engineer profile: linked to 28 knowledge items and 35 skills from ESCO and seven e-competences from EN16234;
  ○ Technician Profile: linked to 20 knowledge items and 19 skills from ESCO and seven e-competences from EN16234.

## 5. Conclusions and Future Lines

This article has explored in more detail two of the aspects linked to human factors, identified as key factors for IoT implementation by the maturity models in the literature: user attitude toward IoT and qualification of the technical implementation team. Our work has focused on the opinion of involved stakeholders in each case:

- In the case of the users’ side, we addressed a specific survey to non-technical managers and professionals in SMEs (as these organizations have fewer resources to work with disrupting technology such as IoT) and educators of future non-technical professionals;
- In the case of the qualification of ICT professionals for IoT solutions, we preferred to have a broad spectrum of opinions, covering the clients’ side (municipality managers and professionals), the providers’ side (technical managers and professionals), and the users’ side. The survey explored the specific details of the recommended qualifications for professionals working in teams where the implementation of IoT (and connected aspects of security and data management) are intensive such as in SC projects. We have shown that SC projects could be representative of the case of general IoT implementations.

Thanks to the analysis of the results of both surveys, we have provided the answer to the two research questions RQ1 and RQ2, adding details to the two human factors linked to them and identified by maturity models for the implementation of IoT. In the case of RQ1, the models already identified that the attitude and culture of IoT users was a key factor for a successful implementation of IoT solutions. In our case, we have confirmed their importance and provided more specific indications: users in Europe consider it essential to address their adaptation to changes caused by IoT with special care regarding possible differences among countries (market conditions, legislation, etc.). They have also listed the set of topics recommended for training and information prior to the implementation of IoT: the adaptation to new contexts created by IoT, the acquisition of basic IoT literacy, changes in business models and in occupation profiles, skills and qualifications, challenges in privacy, security, and legal consequences, and challenges in social aspects.

In the case of RQ2, we have seen how the answer to RQ2 resulted in a specific description of the functions, skills, and knowledge recommended for a good qualification of the technical implementation team, both for the role of engineer and for one of the technicians. Going beyond the mere indication of maturity models regarding “the capabilities of the IoT implementation support team”, the results depict a detailed set of skills and descriptions which can help to better prepare the technical teams for the successful implementation of IoT. The mapping to ESCO will ensure a better understanding and improved alignment with terminology and a classification that it is compulsory in all member states of the European Union since 2021, thus facilitating the adoption across the continent. The mapping to EN16234 also ensures an enhanced connection to ICT industry practices adopted by all types of organizations in Europe, promoting a good understanding of the recommended profiles.

The results have an evident geographical limitation as the samples for surveys were focused on Europe. Although we think that possible differences for developed countries would be minimal, we are planning an additional collection of data from stakeholders in the rest of the world, then allowing for a deeper study of the two human factors involved in our research questions. Possibly, the factor of differences among countries in conditions for IoT already confirmed in the European scenario may possibly be much more relevant with this wider sample.

In the case of RQ2, we approached the study through the context of SC projects, although we have confirmed through different results of our study that stakeholders consider this context as representative of IoT implementations. We are also planning to address additional contexts in our study of the recommended qualification of the technical team for IoT solutions through two actions: expanding the collection of data with a survey to other types of IoT projects and by a compilation of data from the future training activities of technical professionals planned by the project SMACITE after the description of the qualification profiles. New data could help us to verify and refine the qualification guidelines for successful IoT projects.

## Figures and Tables

**Table 1:** Distribution among roles of different categories.

Main Sector (in Bold) and Subsectors	%
**Public sector and authorities (client side)**	**18.52%**
Policy authority or decision maker	5.19%
Municipal city planner or urbanism expert	0%
Municipal technical manager	3.70%
Municipal technician	1.48%
Other	8.15%
**Business sector and providers (supply side)**	**54.81%**
Business manager in IT solutions provider	20%
ICT project manager in solutions provider	14.81%
ICT engineer in solutions provider	9.63%
ICT technician in solutions provider	2.96%
Other	7.41%
**Civil society (user side)**	**26.67%**
Expert in smart cities (academia, research, education, etc.: out-of-solution providers)	13.33%
Representative of citizens’ association	2.96%
Sociologists or similar specialists in urban life	1.48%
Other	8.89

**Table 2:** Functions for engineers.

Category	ESCO Reference for Inspiration and Extraction of Items	Description for Survey
IoT	Smart home engineer (2151.2)	FE1. Design, integration, and acceptance testing of automation systems integrating connected devices and smart appliances within residential facilities. Work with key stakeholders to ensure the desired project outcome including wire design, layout, appearance, and component programming.
Cybersecurity	ICT security engineer (252.9)	FE2. Advise and implement solutions to control access to data and programs and ensure the protection of processes. Responsible for the protection and security of systems and networks and design, plan, and execute the system’s security architecture, with models and security policies and procedures.
Data analytics	Data analyst (2511.3) and data scientist (2511.4)	FE3. Collect and interpret rich data sources, manage large amounts of data, merge sources, ensure consistency, and create visualizations to aid in understanding data using mathematical models and communicate insights and findings to the team and, if required, to non-experts, as well as recommend ways to apply data.
Machine learning and Big Data	No reference occupations: selection of skills and knowledge	Not included in the survey. Preliminary analysis from case studies considers this area as optional in terms of responsibilities.

**Table 3:** Functions for technicians.

Category	ESCO Reference for Inspiration and Extraction of Items	Description for Survey
IoT	Smart home installer (7421.7)	FT1. Install and maintain automation systems, connected devices, and smart appliances at customer sites. Also, act as a user educator and resource for product and service recommendations for customers’ needs for comfort, convenience, security, and safety.
Cybersecurity	ICT security technician (3512.3)	FT2. Propose and implement necessary security updates and measures whenever required. In addition, advice, support, inform, and provide training and security awareness.
Data analytics	Data analyst (2511.3) and data scientist (2511.4)	FT3. Import, clean, validate, model, or interpret collections of data for business goals and given criteria. Also, ensure consistent and reliable data from sources and repositories and prepare reports with visualizations such as graphs, charts, and dashboards.
Machine learning and Big Data	No reference occupations: selection of skills and knowledge	Not included in the survey. Preliminary analysis from case studies considers this area as optional in terms of responsibilities.

**Table 4:** Mapping of functions to the e-competence of EN16234.

Role	e-Competence	Level
Engineer	B.6 (ICT systems engineering)	4
Engineer	E.2 (project and portfolio management)	4
Engineer	A.6 (application design)	3
Engineer	B.4 (solution deployment)	3
Engineer	E.8 (information security management)	4
Engineer	D.7 (data science and analytics)	3
Engineer	B.3 (testing)	3
Technician	E.2 (project and portfolio management)	2
Technician	B.1 (application development)	2
Technician	B.4 (solution deployment)	2
Technician	E.8 (information security management)	3
Technician	D.7 (data science and analytics)	2
Technician	B.4 (solution deployment)	1
Technician	C.1 (user support)	1

**Table 5:** ESCO descriptions for engineers.

Category	ESCO Reference for Inspiration and Extraction of Items	Description for Survey
IoT skills	ESCO skill: “design smart grids”	SE1. Design and calculate smart systems, based on grid load, duration curves, energy simulations, etc.
IoT knowledge	Three ESCO knowledge items: skills “internet of things”, “smart grids systems”, and “building automation”	KE1. Principles, requirements, limitations, and vulnerabilities of smart connected devices and automatic control systems for digital control, distribution saving, and use of energy and information management.
Cybersecurity skills	Nine ESCO skills: “verify formal ICT specifications”, “analyze ICT system”, “identify ICT security risks”, “develop information security strategy”, “ensure information security”, “perform risk analysis”, “define security policies”, “manage disaster recovery plans”, and “implement ICT risk management”	SE2. Create a strategy for safety and security, with a set of rules and policies. Analyze systems to identify risks and implement procedures for identifying, assessing, and mitigating them and prepare recovery plans.
Cybersecurity knowledge	Four ESCO knowledge items: “cyber security”, “ICT security standards”, “risk management”, and “cloud security and compliance”	KE2. Methods and standards to protect ICT systems, resources, and users against illegal or unauthorized use, identifying, assessing, and dealing with all types of risks, including from cloud computing.
Data analytics skills	Five ESCO skills: “Interpret current data”, “apply statistical analysis techniques”, “manage data”, “define data quality criteria”, and “perform data analysis”	SE 3. Define data quality criteria and perform data analysis with statistical techniques to interpret data to assess development and innovation.
Data analytics knowledge	Five ESCO knowledge items: “manage cloud data and storage”, “statistics”, “data models”, “visual presentation techniques”, “unstructured data”	SE4. Statistical methods, practices, and data techniques for collection, organization, the structure of data elements, analysis, interpretation, and presentation of data (local and cloud) to reinforce human understanding.
Machine learning and Big Data skills	Two ESCO skills: “perform data mining” and “analyze big data”	SE4. Explore large datasets to reveal patterns using statistics, databases, or AI and present information in a comprehensible way.
Machine learning and Big Data knowledge	Three ESCO knowledge items: “machine learning”, “data mining”, and “smart city features”	KE4. Big Data technologies (machine learning, data mining, etc.) for smart cities to develop novel software ecosystems upon which advanced mobility functionalities emerge.

**Table 6:** ESCO descriptions for technicians.

Category	ESCO Reference for Inspiration and Extraction of Items	Description for Survey
IoT skills	ESCO skill: “install smart devices”	ST1. Install connected devices, (sensors, light switches, plugs, energy meters, cameras, etc.) and interconnect these devices to the system and to relevant sensors.
IoT knowledge	Three ESCO knowledge items: skills “internet of things”, “smart grids systems”, and “building automation” (same as in KE1)	KT1. Categories, requirements, limitations, and vulnerabilities of smart connected devices and automatic control systems for digital control, distribution, saving, and use of energy and information management (adapted to the technician role).
Cybersecurity skills	Four ESCO skills: “analyze ICT system”, “identify ICT system weaknesses”, “solve ICT system problems”, and “define firewall rules”	ST2. Analyze the functioning and performance of systems to identify and categorize weaknesses and vulnerabilities to intrusions or attacks. Deploy diagnostic tools and resources to solve them, including firewall configuration.
Cybersecurity knowledge	Three ESCO knowledge items: “cyber-attack counter-measures”, “attack vectors”, and “cyber security” (this is common to KE2)	KT2. Methods or pathways deployed by hackers to penetrate or target systems illegally and techniques and tools to detect and avert malicious attacks and protect ICT systems, resources, and users.
Data analytics skills	Four ESCO skills: “perform data cleansing”, “collect ICT data”, “normalize data”, and “manage data” (this is common to SE3)	SE 3. Collect data from connected devices, detect and correct corrupt records from datasets (according to defined quality criteria), and normalize data to minimize dependency, eliminate redundancy, and increase consistency.
Data analytics knowledge	Five ESCO knowledge items: “manage cloud data and storage”, “statistics”, “data models”, “visual presentation techniques”, and “unstructured data” (all the same as in KE3)	KE3. Understanding statistical methods, practices, and data techniques for collection, organization, structuring data elements, analysis, interpretation, and presentation of data (local and cloud) to reinforce the human understanding of information (adapted to the technician role).
Machine learning and Big Data skills	Two ESCO skills: “perform data mining” and “analyze big data” (the same as in SE4)	SE4. Explore large datasets identifying patterns according to predefined methods with statistics, databases, or AI and generate reports of information in a comprehensible way (adapted to the technician role).
Machine learning and Big Data knowledge	Three ESCO knowledge items: “machine learning”, “data mining”, and “smart city features” (the same as in ST4)	KE4. Principles, methods, and algorithms of machine learning, statistics, and data mining (adapted to the technician role).

## Data Availability

The data presented in this study are openly available in Zenodo at 10.5281/zenodo.7492255.
